# Delay-Dependent Impairments in Memory and Motor Functions After Acute Methadone Overdose in Rats

**DOI:** 10.3389/fphar.2018.01023

**Published:** 2018-09-10

**Authors:** Leila Ahmad-Molaei, Hossein Hassanian-Moghaddam, Fariba Farnaghi, Carlos Tomaz, Abbas Haghparast

**Affiliations:** ^1^Neuroscience Research Center, School of Medicine, Shahid Beheshti University of Medical Sciences, Tehran, Iran; ^2^Department of Clinical Toxicology, Loghman-Hakim Hospital, School of Medicine, Shahid Beheshti University of Medical Sciences, Tehran, Iran; ^3^Social Determinants of Health Research Center, Shahid Beheshti University of Medical Sciences, Tehran, Iran; ^4^Department of Pediatric Clinical Toxicology, Loghman-Hakim Hospital, School of Medicine, Shahid Beheshti University of Medical Sciences, Tehran, Iran; ^5^Neuroscience Research Program, CEUMA University, São Luís, Brazil

**Keywords:** methadone, naloxone, learning and memory performance, motor coordination, overdose, rat

## Abstract

Methadone is used as a substitution drug for the treatment of opioid dependence and chronic pain. Despite its widespread use and availability, there is a serious concern with respect to the relative safety of methadone. The purpose of this study was to characterize how acute methadone overdose affects the cognitive and motor performance of naïve healthy rats. The methadone overdose was induced by administering an acute toxic dose of methadone (15 mg/kg; ip; the equivalent dose of 80% of LD_50_) to adolescent rats. Resuscitation using a ventilator pump along with a single dose of naloxone (2 mg/kg; ip) was administered following the occurrence of apnea. The animals which were successfully resuscitated divided randomly into three apnea groups that evaluated either on day 1, 5, or 10 post-resuscitation (M/N-Day 1, M/N-Day 5, and M/N-Day 10 groups) in the Y-maze and novel object memory recognition tasks as well as pole and rotarod tests. The data revealed that a single toxic dose of methadone had an adverse effect on spontaneous behavior. In addition, Recognition memory impairment was observed in the M/N-Day 1, 5, and 10 groups after methadone-induced apnea. Further, descending time in the M/N-Day 5 group increased significantly in comparison with its respective Saline control group. The overall results indicate that acute methadone-overdose-induced apnea produced delay-dependent cognitive and motor impairment. We suggest that methadone poisoning should be considered as a possible cause of delayed neurological disorders, which might be transient, in some types of memory or motor performance in naïve healthy rats.

## Introduction

Methadone is a long-acting, synthetic mu-opioid agonist having multiple actions and pharmacologic properties that are similar to morphine (Barbosa Neto et al., 2015). Methadone has long been used for the treatment of opioid dependence and detoxification or maintenance in cases of opioid addiction because of its long efficacy and low cost (Kleber, 2007). In addition, like other opioids such as buprenorphine, fentanyl, morphine, and oxycodone, methadone is used to alleviate severe pain (Argoff and Silvershein, 2009). Despite its considerable therapeutic applications, acute methadone intoxication may lead to morbidity and death (Shields et al., 2007; Soltaninejad et al., 2014). In the United States, opioid drugs were involved in 61% of all drug overdose deaths and caused more than 28,000 deaths in 2014 (Rudd et al., 2016). Acute poisoning with methadone continues to occur after therapeutic, recreational or accidental use (Jones et al., 2012). In Iran, opium was the drug of choice in 50% of all drug abuse from 2006 to 2009, but the prevalence of methadone toxicity has increased significantly from 2.26% in 2006 to 24.72% in 2011 (Hassanian-Moghaddam et al., 2014).

Since the opiate naïve patients have no tolerance to opiates, (Drummer et al., 1992; Milroy and Forrest, 2000) the stabilization phase should be carefully assessed to reduce the risk of overdose during the induction period to avert the risk of toxicity and death in methadone maintenance treatment (MMT) programs (Morgan et al., 2006; Modesto-Lowe et al., 2010). Several studies indicate a 10-fold increase in methadone-induced toxicity and related death after the increase in the number of methadone maintenance clinics and its arbitrary consumption in recent decades (Shields et al., 2007; Graham et al., 2008). The incidence of poisoning with methadone in children is common due to the availability of this drug as used by family members (Pragst et al., 2013; Shadnia et al., 2013). Methadone poisoning should be considered as a serious threat to naïve, healthy subjects, especially children, as very low doses can cause severe complications or death due to its toxicity (Modesto-Lowe et al., 2010; Jabbehdari et al., 2013; Hassanian-Moghaddam et al., 2017). Indeed, some studies have associated therapeutic doses of methadone with the occurrence of sudden death due to respiratory apnea or cardiac arrest (Chugh et al., 2008).

Few studies have examined cognitive and sensorimotor performance after an acute dose of methadone-induced toxicity in clinical or experimental trials in healthy volunteers. Most studies have examined the effect of the prolonged use of methadone, which can result in neuropsychological impairment as compared to opioid-naïve, healthy controls (Prosser et al., 2006). There is considerable evidence that chronic exposure to methadone in animals can have an adverse effect on memory processes (Hepner et al., 2002; Verdejo et al., 2005). Moreover, patients undergoing the MMT program usually experience limited short-term memory and deficits in working memory (Sjøgren et al., 2000; Mintzer and Stitzer, 2002), visuospatial attention, long-term memory (Prosser et al., 2006) and general cognitive speed (Mintzer et al., 2005) which are in part due to white matter abnormalities (Lin et al., 2012). It has been shown that acute administration of methadone impairs sensorimotor abilities and memory retrieval in rats (Tramullas et al., 2007). Because it has a significantly long half-life of 25–52 h, even a single acute administration of methadone can cause delayed clinical manifestations, including respiratory depression, apnea and unexpected death (LoVecchio et al., 2007).

Despite the fact that, in recent years, methadone overdose has increased, little data is available about the adverse manifestations of methadone overdose in experimentally naïve animals. In addition, behavioral research in human subjects is extremely rare because of ethical considerations. The present study aimed to investigate whether or not a single toxic dose of methadone will result in apnea-caused impairment on cognitive and/or motor functions in adolescent rats.

## Materials and Methods

### Animal

One-month- old male Wistar rats, (Pasteur Institute, Tehran, Iran) weighting 50-80 g, were kept under the standard laboratory conditions (22°C, 12-h light/12-h dark cycle) and randomly allocated to different experimental groups. All rats habituated to their new environment for 5 days before the experimental procedure started. The tests were performed between 8:00 and 16:00 h. All procedures were conducted according to the Guide for the care and use of laboratory animals (National Institutes of Health Publication No. 80–23, revised 1996) and were approved by the Research and Ethics Committee of School of Medicine, Shahid Beheshti University of Medical Sciences (IR.SBMU.MSP.REC.1395.33), Tehran, Iran.

### Drugs

In the present study, Methadone hydrochloride 5 mg/ml (Darou-Pakhsh Pharmaceutical Company, Tehran, Iran) and Naloxone 0.4 mg/ml (Tolid-Darou Pharmaceutical Company, Tehran, Iran) were used.

### Experimental Design and Drug Administration

**FIGURE 1:**
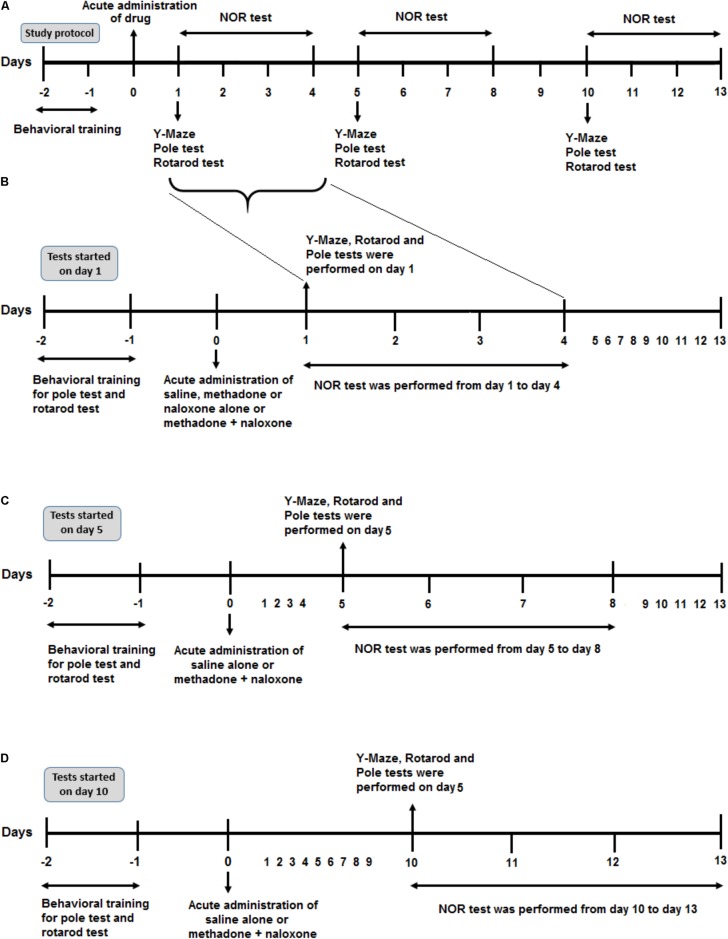
Schematic illustration of the experimental schedule. **(A)** Protocol overview of the study. After 2 days of behavioral training (rotarod and pole tasks), all rats were administered drug injection on day 0. According to the time of the behavioral test, (starting on day 1, 5, or day 10 after drug administration) they represented here in three parts. **(B)** After drug application, six separated animal groups (Saline, Methadone, Naloxone, M/N-Sedate, Saline control-Day 1, and M/N-Day 1) were used to evaluate different behavioral tests including Y-Maze, Rotarod test and Pole test on day 1 after drug administration and Novel object recognition (NOR) test from day 1 to day 4 post-treatment. **(C)** In two other separated groups (Saline control-Day 5, M/N-Day 5), after administration of saline alone or methadone + naloxone on day 0, Y-Maze, Pole test, and Rotarod test were carried out on day 5 followed by NOR test from day 5 to day 8. **(D)** In Saline control-Day 10 and M/N-Day 10 groups, 10 days after administration of saline alone or methadone + naloxone, Y-Maze, Pole test and Rotarod test were carried out on day 10 followed by NOR test from day 10 to day 13.

In order to induce acute methadone overdose, rats intraperitoneally (i.p.) received a single toxic dose of 15 mg/kg of methadone at equivalent doses (80% of the LD50) which was chosen based on Chevillard study (Chevillard et al., 2009). Slow and difficult breathing, dizziness, cold and clammy skin, motionlessness, drowsiness, straub tail, muscular rigidity, plantar cyanosis, and irritability were seen after the administration of a single toxic dose of methadone in adolescent rats. It has been noted that the primary signs of opioid intoxication include: pinpoint pupils, respiratory depression, and confusion/unconsciousness, referred to as the opioid overdose triad (Ford, 2001). In 35% of all rats, methadone- induced apnea and caused death if they were left untreated, but the rest of the animals regained normal respiration rate after a few hours without any intervention which was randomly selected as methadone group. In order to evaluate cognitive and motor functions in the rats which experienced apnea (cessation of respiration was for 20 s) (Gaspari and Paydarfar, 2007), an acute single dose of naloxone (2 mg/kg; i.p.) (Farahmandfar et al., 2010; Zamani et al., 2015) was administered following methadone-induced apnea. In addition, resuscitation procedure was performed by a respirator pump to serve artificial respiration (Model V5KG, Narco-Biosystems Inc., Houston, TX, United States). Naloxone administration which was done concomitantly with resuscitation, recovered apnea in 67% of all rats in which had cessation of respiration for 20 s. Therefore, animals which have been successfully resuscitated, were randomly divided into three groups so-called “M/N” groups (rats which received naloxone after methadone overdose) to measure neurological functions either on day 1st, 5th, or 10th post- resuscitation (including M/N-Day 1, M/N-Day 5, and M/N-Day 10 groups; **Figure 1**). In other groups all behavioral tests were carried out only 1 day after the drug administration. Methadone group was selected randomly from the animals which re-obtained normal respiration rate after administration of a single toxic dose of methadone without any intervention. There was another group without apnea (M/N-Sedate) in which they received the same dose of methadone but naloxone administration was delivered at the beginning of the sedation state. Naloxone group with a single administration dose was designated to test memory and motor functions exclusively in a separated group. Control (Saline) group received an equal volume of saline 0.9% and behavioral assessment was performed 1 day after saline injection. It has been noted that data in the M/N-Day 1, 5 and 10 groups were compared with the Saline control-Day 1, Day 5 and Day 10 groups, respectively. Each group consisted of 6–14 rats while were grouped in 10 experimental groups.

### Behavioral Training

For five consecutive days, rats were handled for 5 min before starting any test procedure. All rats had multiple behavioral tests including the Y-maze, novel object memory recognition (NOR) tests as well as pole, rotarod tasks to investigate neurological functions. Animal behaviors were observed by a researcher who was blind to the experimental groups. The order of tests was the same for all animals. In order to avoid the effect of any confounding factors or minimize the influence of stress on animals for each task, the order of behavioral tests was as follow; (1) Y-maze test, (2) NOR test, (3) pole test, and (4) rotarod test. In addition, locomotor activity was measured for each rat during a 5-min period on the test day (**Figure 6**).

### Spontaneous Alternation Behavior Test (Y-Maze)

The Y-maze test can be used as a measure of spatial working memory in rodents. It is applied to evaluate the natural tendency of animals to explore new places by recording spontaneous alternation behavior. In this study, the Y-maze apparatus consisted of the Y-shaped maze with three identical arms at 120 degrees to each other which was made of gray- painted Plexiglass. Rats were placed at the end of the one arm and allowed to navigate the maze during an 8-min trial. The sequence and number of the total arm entries were manually registered. An arm entry was defined when four paws were within the arm. An alternation behavior was determined from consecutive entries into the three different arms. The percentage of alternation was calculated as the following equation:

{(number of alternation)/(total number of arm entries-2)}×100

Total number of arm entries were recorded as well. In addition, animals with 8 arm entries or less were omitted from analysis during an 8-min session (Holcomb et al., 1998; Ma et al., 2007; Farhadinasab et al., 2009).

### Novel Object Recognition Test (NOR)

The task procedure consisted of three distinct phases: habituation, familiarization, and test. The NOR task was performed to measure non-spatial memory. An open field box (40 × 40 × 40 cm) (length × width × height) was made of black wood used as an apparatus to test recognition memory. Rats were allowed 1 h of accustomed to the test room before starting each phase. All rats were given a 10-min session to explore apparatus with no objects as a habituation phase in two consecutive days. During the familiarization phase, two identical objects (A1 and A2) were attached to the floor at an equal distance, 10 cm from the walls while positioned in the two adjacent corners. Each rat was placed in the box facing the wall opposite the two identical objects allowed to explore freely for 3 min. If the total exploration time was less than 12 s for the novel and familiar objects during familiarization phase, the rat excluded from the data analysis. Then, object A1 or A2 was replaced with object B before starting the test phase. Evaluation of short-term memory was conducted 90-min later in which the familiar object and the new object (object B) located in the open field. Rats were allowed to explore freely for 3-min in the box. After 24 h, object B was replaced with object C for testing long-term memory in a 3-min period to explore the box. The time spent exploring both objects (familiar and novel) was recorded by a video tracking system. The preference index was calculated as the exploration time for the novel and familiar objects relative to the total time (Antunes and Biala, 2012; Cohen and Stackman, 2015).

### Pole Test

Pole test was first introduced by Ogawa (Ogawa et al., 1985) to evaluate movement impairment and coordination in mice indicating a practical task for the basal ganglia dysfunction. The apparatus consisted of a 90 cm vertical wooden pole length and 5 cm in diameter which covered with the rough surface that led into their home cage. All animals received training sessions on two consecutive days (10-trials/day) before the test day where they were placed with the head facing upward right below the top. During the first trial on the first day, if the rat failed to climb down, it was gently turned around on the pole and thus forced to return to its home cage. On the test day, three parameters were measured; t-turn (the time to turn downward), descending time (the time to descend the pole) and total time (the time to turn downward and descent the pole to reach the floor). When the animal failed to turn downward after 120 s, it was taken as a default value. The animals were tested on 3 trials on the test day and the average time was used as the pole test score.

### Rotarod Test

Rotarod apparatus is used to evaluate motor coordination and skills in rodents (Dunham and Miya, 1957; Deacon, 2013). Animals were placed on a 2.5 cm diameter drum supported 25 cm above the base of the apparatus. Rats were trained 5 trials a day, separated by 30 min inter-trial intervals on the two successive days. Animals were placed in the testing room for 1 h before starting the test to acclimate to the testing. The rats were held by their tails while facing away from the direction of rotation the drum such that animals released on the horizontal rod while walking forward to keep their balance. The Rotation speed was set at 20 rpm in the training and testing sessions. If the rat failed to grasp rod properly and fell before 5 s, the procedure would start again to keep the balance. During the test session, animals were assessed by placing on the rod until either they fell off or reached a maximum 300 s. The mean values of the 3 test trials were calculated for each rat.

### Locomotor Activity

Total numbers of infrared beam break automatically were recorded. Rats were placed in a box (40 × 40 × 40 cm) to evaluate locomotion. Locomotor activity was tracked by a 5 × 5 photobeam configuration for each rat in which sensed infrared beam interruption caused by movement of the animal in real time for 5 min (Zhang and Kong, 2017).

### Statistical Analysis

All data were represented as mean ± SEM (standard error of mean) and were analyzed by commercially available software GraphPad Prism^®^ 5.0. In order to compare data between two groups in familiarization phase in NOR, data in apnea groups which have been compared with their respective Saline control groups, paired or unpaired *t*-test were used, respectively. For multiple comparisons between groups, one-way analysis of variance (ANOVA) followed by *post hoc* Newman–Keuls test was applied as needed. The level of statistical significance was set at *P*-value less than 0.05 (*P* < 0.05).

## Results

### Effect of a Single Acute Toxic Dose of Methadone Administration on Spatial Working Memory in the Y-Maze Test

**FIGURE 2:**
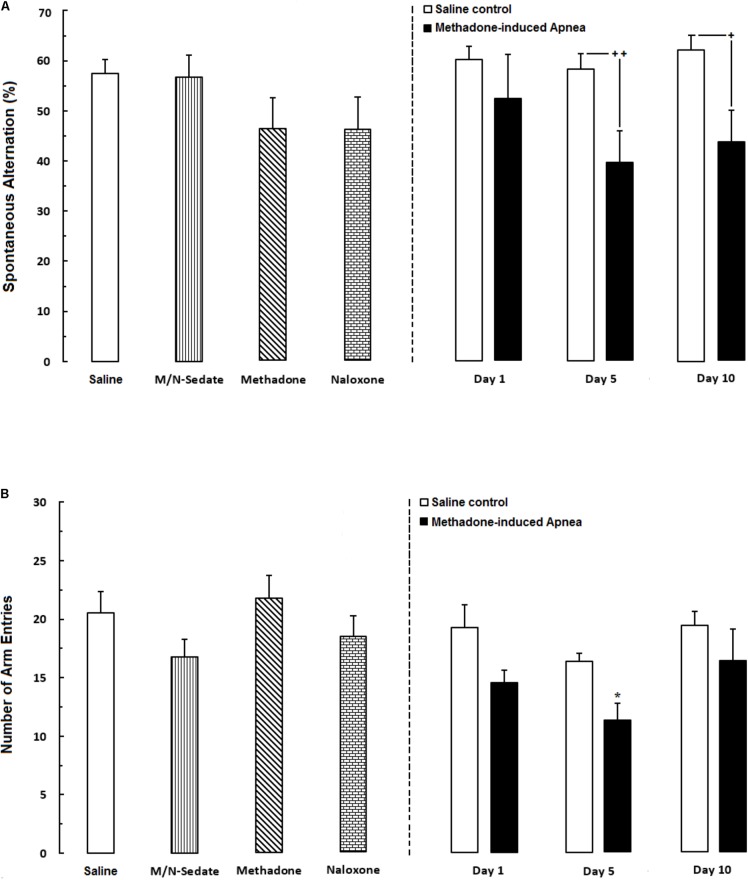
**(A)** Spontaneous alternation behavior and **(B)** Total number of arm entries were recorded in different groups, including; M/N-treated groups (a single dose of naloxone was administered after methadone overdose in apnea stage, in animals which experienced apnea and spontaneous alternation behavior was evaluated either on day 1, 5, or 10 day post-resuscitation; M/N-Day 1 (*n* = 6), M/N-Day 5 (*n* = 6) and M/N-Day 10 (*n* = 6) groups and their respective Saline control groups; *n* = 6 right panel), M/N-Sedate (*n* = 8; a single dose of naloxone was administered following methadone overdose, immediately in the initial stage of sedation, so behavioral evaluation was carried out only 1 day after drug administration), Saline; *n* = 12, methadone; *n* = 8 and naloxone; *n* = 7 (saline, methadone or naloxone were administered alone in separated groups in which spontaneous alternation behavior and the number of arm entries were recorded only 1 day after drug administration during an 8-min trial in adolescent rats) groups. Animals received methadone (15 mg/kg; i.p.) or naloxone (2 mg/kg; i.p.) alone or both (apnea groups) in a single dose. Each bar shows the mean ± SEM for 6–12. ^∗^*P* < 0.05 different from the Saline group. **^+^***P* < 0.05 and **^++^***P* < 0.01 different from their respective Saline control groups.

As shown in **Figure 2A**, unpaired *t*-test analysis revealed that administration of an acute toxic dose of methadone (15 mg/kg; i.p.) which caused apnea and subsequent naloxone injection (2 mg/kg; i.p.), impaired the percentage of the spontaneous alternation behavior in the M/N-Day 5 [*t*(9) = 2.908, *P* < 0.01] and M/N-Day 10 groups [*t*(10) = 2.695, *P* = 0.0225] when compared with their respective Saline control groups (right panel) while this parameter was not different in the M/N-Day 1 as compared to its Saline control group [*t*(12) = 0.9745, *P* = 0.3491; ns]. As depicted in **Figure 2A**, one way ANOVA followed by Newman–Keuls *post hoc* analysis showed that there was not significant deficient in the alternation behavior in M/N-Sedate, M/N-treated rats (with apnea, right panel), methadone (without apnea) or naloxone groups as compared with the Saline [*F*_(6,46)_ = 1.571, *P* = 0.1773] group. Moreover, in **Figure 2B**, unpaired *t*-test analysis manifested that the number of arm entries were not different in the M/N-Day 1 [*t*(12) = 1.947, *P* = 0.0754; ns], Day 5 [*t*(9) = 0.8357, *P* = 0.4249; ns] and Day 10 [*t*(10) = 1.010, *P* = 0.3363; ns] groups as compared with their respective Saline control groups (right panel). In addition, One-way ANOVA followed by Newman–Keuls *post hoc* analysis revealed no significant reduction in the number of total arm entries in the M/N-Sedate, as well as M/N-Day 1 and Day 10 groups (with apnea, right panel), methadone or naloxone groups when compared with the Saline group [*F*_(6,47)_ = 2.232, *P* < 0.05] but not for the M/N-Day 5 groups which showed significant reduction as compared with the Saline group.

### Effect of an Acute Toxic Dose of Methadone Administration in Recognition Memory in Adolescent Rats

**FIGURE 3:**
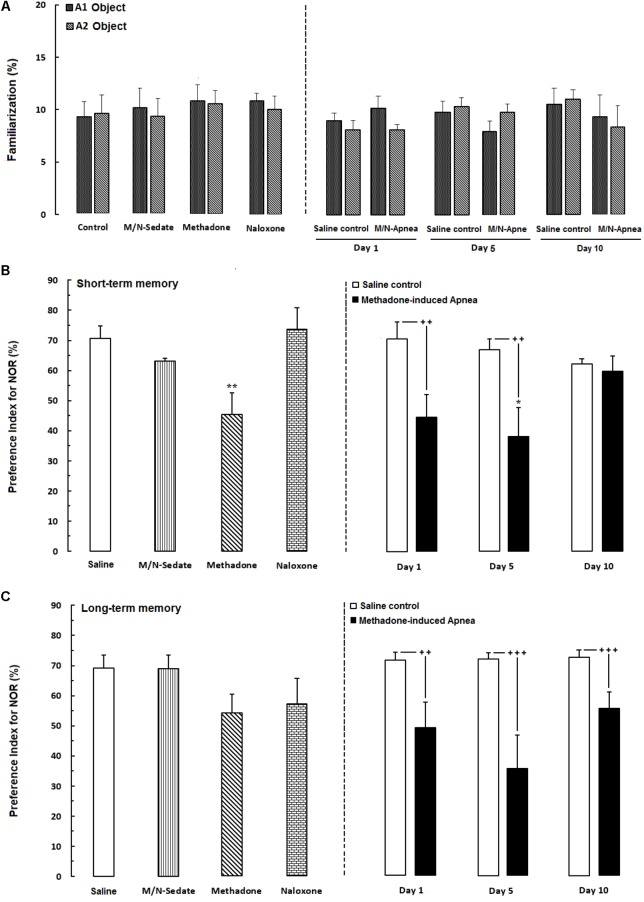
Performance of recognition memory in the novel object recognition task in three sessions as follow; **(A)** Familiarization (rats were allowed to explore freely two identical objects A1 and A2 for 3-min), **(B)** Short-term memory (object A1 or A2 was replaced with object B while rats were allowed to explore for 3-min), **(C)** long-term memory (object B was replaced with object C which provided rats explored freely two objects for 3-min) in different groups, including; M/N-treated groups (a single dose of naloxone was administered after methadone overdose in apnea stage, in animals which experienced apnea and recognition memory was evaluated either on day 1, 5, or 10 day post-resuscitation; M/N-Day 1 (*n* = 7), M/N-Day 5 (*n* = 6) and M/N-Day 10 (*n* = 6) groups and their respective Saline control groups (*n* = 6); right panel), M/N-Sedate (*n* = 7; a single dose of naloxone was administered following methadone overdose, immediately in the initial stage of sedation, so behavioral evaluation was carried out only 1 day after drug administration), Saline; *n* = 10, methadone; *n* = 11, naloxone; *n* = 8 (Saline, methadone or naloxone were administered alone in separated groups which recognition memory was evaluated only 1 day after the drug administration in adolescent rats) groups. Animals received methadone (15 mg/kg; i.p.) or naloxone (2 mg/kg; i.p.) alone or both in a single dose. Each bar shows the mean ± SEM for 6–11. ^∗^*P* < 0.05 and ^∗∗^*P* < 0.01 different from the Saline group. **^++^***P* < 0.01 and **^+++^***P* < 0.001 different from their respective Saline control groups.

The novel object recognition task is the ability to distinguish the novel from familiar stimuli which is directly dependent on the prefrontal cortex and hippocampus function (Banks et al., 2012; Pezze et al., 2017). In **Figure 3A**, the data obtained analyzed using paired *t*-test exhibited that animals spent equal time to explore both object A1 and A2 and there were not any significant preference in exploring two objects in familiarization phase in the M/N-Day 1 [*t*(6) = 0.7381, *P* = 0.4883; ns], Day 5 [*t*(6) = 0.5558, *P* = 0.5984; ns] and Day 10 [*t*(6) = 0.5176, *P* = 0.6109; ns] groups as compared with their respective Saline control groups (right panel). In **Figure 3B** which shown short-term memory phase, unpaired *t*-test analysis indicated that a single toxic dose of methadone (apnea groups) significantly impaired recognition memory in the M/N-Day 1 [*t*(12) = 2.785, *P* < 0.01] and M/N-Day 5 [*t*(9) = 3.032, *P* < 0.01] when compared with their respective Saline control groups (right panel). One-way ANOVA followed by Newman–Keuls *post hoc* test exhibited that administration of an acute toxic dose of methadone (15 mg/kg; i.p.) with subsequent naloxone (2 mg/kg; i.p.) administration in sedation state (M/N-Sedate group), three M/N-treated groups, as well as methadone and naloxone groups did not have attenuating effects on short-term memory when compared to the Saline group [*F*_(6,46)_ = 3.871, *P* = 0.0010], while this parameter shown significant reduction in the M/N-Day 5 groups as compared with the Saline group. In **Figure 3C**, the data obtained for long-term memory test revealed detrimental effect of methadone overdose on long-term memory in the M/N-Day 1 [*t*(11) = 3.903, *P* = 0.0025] and Day-5 [*t*(9) = 4.512, *P* < 0.001] groups which have been continued on day 13 in M/N-Day 10 group [*t*(11) = 4.285, *P* < 0.001] when compared with their respective Saline control groups (right panel). Moreover, administration of an acute toxic dose of methadone (15 mg/kg; i.p.) or naloxone (2 mg/kg; i.p.) alone as well as M/N-treated rats (right panel) and M/N-Sedate groups did not show any significant deficit in long-term memory when compared to the Saline group [*F*_(6,46)_ = 2.384, *P* = 0.0434].

### Effect of an Acute Toxic Dose of Methadone Administration on Motor Functions in Pole Test in Adolescent Rats

**FIGURE 4:**
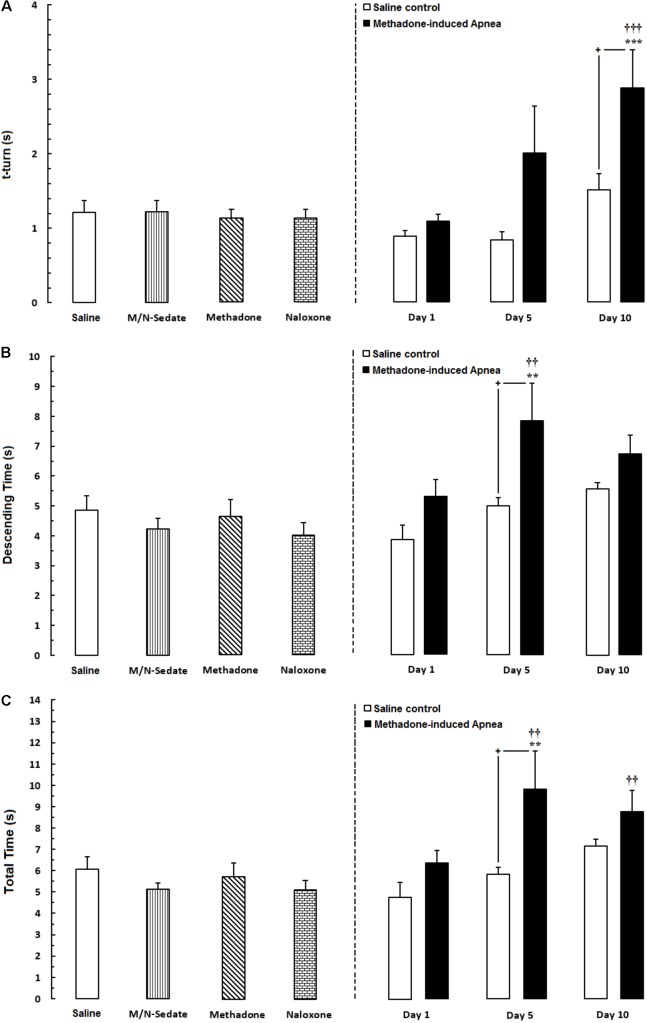
Evaluation of motor performance in pole test such that three parameters were measured including; **(A)** time to turn downward (t-turn), **(B)** descending time (time to move downward to reach the floor and **(C)** total time (time to turn and descending the pole to reach the floor) in different groups, including; M/N-treated groups (a single dose of naloxone was administered after methadone overdose in apnea stage, in animals which experienced apnea and motor behavior was evaluated either on day 1, 5, or 10 day post-resuscitation; M/N-Day 1 (*n* = 11), M/N-Day 5 (*n* = 6) and M/N-Day 10 (*n* = 7) groups and their respective Saline control groups; *n* = 6; right panel), M/N-Sedate; *n* = 9 (a single dose of naloxone was administered following methadone overdose, immediately in the initial stage of sedation, so behavioral evaluation was carried out only 1 day after drug administration), Saline; *n* = 14, methadone; *n* = 8 and naloxone; *n* = 8 (saline, methadone or naloxone were administered alone in separated groups in which motor function was evaluated 1 day after drug administration during pole test in adolescent rats) groups. Animals received methadone (15 mg/kg; i.p.) or naloxone (2 mg/kg; i.p.) alone or both in a single dose. Each bar shows the mean ± SEM for 6-14. ^∗∗^*P* < 0.01 and ^∗∗∗^*P* < 0.001 different from the Saline group. ^††^*P* < 0.01 and ^†††^*P* < 0.001 different from the M/N-Sedate group. **^+^***P* < 0.05 different from their respective Saline control groups.

As exhibited in **Figure 4A** (right panel), unpaired *t*-test analysis showed that there was no significant difference in t-turn between the M/N-Day 1 [*t*(18) = 1.674, *P* = 0.1115; ns], Day 5 [*t*(10) = 1.820, *P* = 0.0988; ns] and their respective Saline control groups but this parameter increased in the M/N-Day 10 group as compared with its respective Saline control group [*t*(12) = 2.180, *P* < 0.05]. Moreover, one-way ANOVA followed by Newman–Keuls *post hoc* test showed that in the M/N-Sedate, methadone and naloxone groups no change has been observed in *t*-turn values when compared with the Saline group [*F*_(6,57)_ = 6.007, *P* < 0.0001] but significant increase revealed in the M/N-Day 10 when compared with the Saline or the M/N-Sedate groups. In **Figure 4B** (right panel), unpaired *t*-test analysis indicated that there was no significant difference in descending time in the M/N-Day 1 [*t*(18) = 1.793, *P* = 0.0898; ns] and Day 10 [*t*(11) = 1.442, *P* = 0.1772; ns] groups as compared with their respective Saline control groups, but in the M/N-Day 5 group, the impairment was obvious in descending time when compared with its respective Saline control group [*t*(10) = 2.209, *P* < 0.05]. However, as shown in **Figure 4B**, one way ANOVA revealed that in the M/N-Sedate, the M/N-Day 1 and Day 10 groups as well as methadone (without apnea) and naloxone groups, motor functions were not impaired as compared with the Saline group [*F*_(6,56)_ = 4.221, *P* = 0.0014] but significant increase in descending time was observed in the M/N-Day 5 when compared with the Saline or M/N-Sedate groups. Additionally, in **Figure 4C** (right panel), unpaired *t*-test analysis showed that there was no significant impairment in motor function in the M/N-Day 1 [*t*(18) = 2.008, *P* = 0.0599; ns] and Day 10 [*t*(12) = 1.307, *P* = 0.2155; ns] groups as compared with their respective Saline control groups, but in the M/N-Day 5, the detrimental effect of methadone overdose was seen in motor activity when compared with its respective Saline control group [*t*(10) = 2.217, *P* < 0.05]. Furthermore, As depicted in **Figure 3C**, One-way ANOVA followed by Newman–Keuls *post hoc* test revealed that motor function did not impair in the M/N-Sedate as well as in methadone and naloxone groups as compared with the Saline group [*F*_(6,56)_ = 4.605, *P* = 0.0007] but total time increased significantly in the M/N-Day 5 and Day 10 groups in comparison with the Saline or M/N-Sedate groups.

### Rotarod Test

**FIGURE 5:**
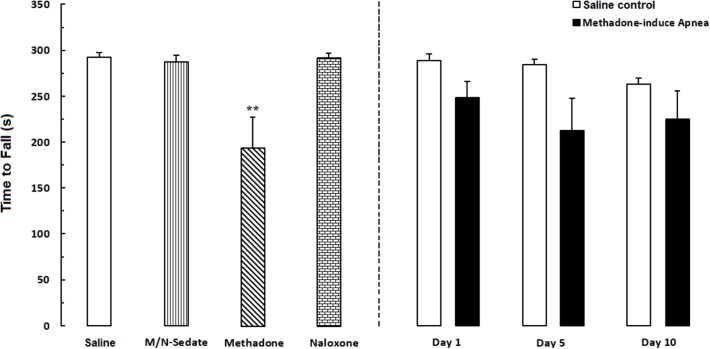
Evaluation of motor coordination and balance in rotarod test during a 5-min trial in different groups, including; M/N-treated groups (a single dose of naloxone was administered after methadone overdose in apnea stage, in animals which experienced apnea and motor coordination was evaluated either on day 1, 5, or 10 day post-resuscitation; M/N-Day 1 (*n* = 9), M/N-Day 5 (*n* = 6) and M/N-Day 10 (*n* = 8) groups and their respective Saline control groups; *n* = 6; right panel), M/N-Sedate (*n* = 8; a single dose of naloxone was administered following methadone overdose, immediately in the initial stage of sedation, so behavioral evaluation was carried out only 1 day after drug administration), Saline; *n* = 10, methadone; *n* = 9 and naloxone; *n* = 6 (saline, methadone or naloxone was administered alone in separated groups in which coordination assessment was evaluated only 24 h following the drug administration in a 5-min trial in adolescent rats) groups. Animals received methadone (15 mg/kg; i.p.) or naloxone (2 mg/kg; i.p.) alone or both in a single dose. The time each rat stay on the rod before falling was recorded. Each bar shows the mean ± SEM for 6–10. ^∗∗^*P* < 0.01 different from the Saline group.

Unpaired *t*-test analysis indicated that there were no significant detrimental effect of methadone overdose on motor coordination in rotarod test in the M/N-Day 1 [*t*(14) = 1.915, *P* = 0.0762; ns], Day 5 [*t*(11) = 2.153, *P* = 0.0543; ns] and Day 10 [*t*(13) = 1.689, *P* = 0.1150; ns] groups when compared with their respective Saline control groups (right panel). In addition, One-way ANOVA with Newman–Keuls *post hoc* test showed that in methadone group (without apnea) there was significant impairment in motor coordination as compared with the Saline group [*F*_(6,49)_ = 3.386, *P* = 0.0071]. However, in M/N-Sedate and naloxone groups as well as three M/N-treated groups, no significant differences revealed on motor coordination when compared with the Saline group (**Figure 5**).

### Effect of Acute Toxic Dose of Methadone on Locomotor Activity

**FIGURE 6:**
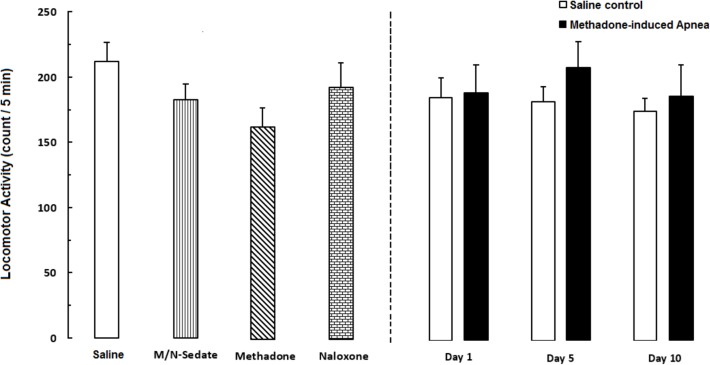
Locomotor activity was recorded by using photobeam activity system during a 5-min session in different groups, including; M/N-treated groups (a single dose of naloxone was administered after methadone overdose in apnea stage, in animals which experienced apnea and locomotor activity was evaluated either on day 1, 5, or 10 day post-resuscitation; M/N-Day 1 (*n* = 10), M/N-Day 5 (*n* = 6) and M/N-Day 10 (*n* = 6) groups and their respective Saline control groups; *n* = 6; right panel), M/N-Sedate (*n* = 11; a single dose of naloxone was administered after methadone overdose immediately in the initial stage of sedation state, so behavioral evaluation was carried out only 1 day after drug administration), Saline; *n* = 14, methadone; *n* = 12 and naloxone; *n* = 8 (saline, methadone or naloxone was administered alone in separated groups in which locomotor activity was evaluated 1 day after drug administration) groups. Animals received methadone (15 mg/kg; i.p.) or naloxone (2 mg/kg; i.p.) alone or both in a single dose. Administration of toxic dose of methadone did not change locomotor activity in all groups. Each bar shows the mean ± SEM for 6–14.

As shown in **Figure 6**, unpaired *t*-test analysis showed that a single dose of naloxone (2 mg/kg; i.p.) administration following methadone overdose (15 mg/kg; i.p.) in animals which experienced apnea did not displayed significant deficit in locomotor activity in the M/N-Day 1 [*t*(15) = 0.3083, *P* = 0.7621; ns], Day 5 [*t*(11) = 0.6218, *P* = 0.5468; ns] and Day 10 [*t*(10) = 0.0126, *P* = 0.9902; ns] groups when compared to their respective Saline control groups (right panel). In addition, One-way ANOVA by Newman–Keuls *post hoc* analysis showed that there was no significant difference in locomotor activity in the M/N-Sedate group, M/N-treated rats (right panel, with apnea) as well as methadone and naloxone groups when compared with the Saline group [*F*_(6,62)_ = 1.084, *P* = 0.3817].

## Discussion

The purpose of this research was to investigate the cognitive and motor effects of a single toxic dose of methadone on three random groups of naïve adolescent rats tested on either day 1, 5, and 10 after drug administration as depicted in **Figure 1**. The findings showed that (i) Administration of an acutely toxic dose of methadone induced apnea in 35% of treated rats, (ii) Naloxone as a non-specific opioid receptor antagonist resuscitated 67% of the rats which experienced apnea, (iii) Delay-dependent impairment in cognitive and motor functions was observed in different behavioral tests, (iv) Transient motor impairment following methadone-induced apnea, and (v) Motor deficient in descending time on day 5 after administration of an acute toxic dose of methadone overdose in pole test was observed.

It has been documented that opioids suppress respiration in humans and animals (Van Der Schier et al., 2014). Methadone is a long-acting opioid agonist used for therapy and as medication for abuse/dependence and to treat severe refractory cancer pain (Leppert, 2009; Keane, 2013; Schuckit, 2016). The extensive prescription of methadone has enhanced the risk of life-threatening overdoses in different countries (Paulozzi et al., 2006; Rudd et al., 2016). Buprenorphine, like methadone, is used in the treatment of opioid addiction, but as a partial agonist, displays a ceiling effect; after a certain point, an increase in the dosage will not enhance its effects (Dahan et al., 2006). In contrast, methadone is a full opioid agonist which has the potential to be abused, misused or used non-medically, making overdose-related death, especially due to respiratory depression, a big problem (Ayatollahi et al., 2011; Whelan and Remski, 2012; Soltaninejad et al., 2014). In recent years, methadone has been extensively prescribed in the MMT programs or to relieve pain, giving rise to methadone overdoses by adults using supratherapeutic amounts or by accidental ingestion in the pediatric population (Soltaninejad et al., 2014).

In the Y-maze task, which is a measure of spatial working memory, impairment was revealed in the alternation behavior after methadone overdose. Consistent with our results, Hepner et al. (2002) indicated that methadone which acutely administered impaired the working memory version of Morris water task in rats. In the current study, we did not measure the concentration of methadone in blood or brain tissues, but Andersen et al. (2011) indicated that no methadone detected in brain tissue on the test day which showed memory impairment after the drug administration. The long-lasting impairment in learning or memory after acute or chronic opioid administration might be associated with the persistent impairment of different brain functions through several mechanisms, including changes in central signaling proteins (Lou et al., 1999), activation of apoptosis signaling pathway (Emeterio et al., 2006) or impaired synaptic plasticity (Pu et al., 2002). Previous results suggest that the hippocampus and prefrontal cortex are involved in the NOR task (Banks et al., 2012; Pezze et al., 2017). The current findings indicate impairment of short-term memory in the NOR task on days 3, 7, and 12 (timeline was shown in **Figure 1**) after methadone overdose. In addition, the reduced recognition memory in the methadone (without apnea) group and the M/N groups which experienced apnea might be due to the activation or changes in protein signaling or the apoptosis pathways related to methadone overdose which have been induced by a high-dose application of opioids (Tramullas et al., 2007; Andersen et al., 2012). It has been proposed that memory impairment might be the result of the direct toxicity of methadone that overstimulates the opioid receptors in the hippocampus and limbic system related to particular forms of learning and memory, including spontaneous object recognition memory (Pertschuk and Sher, 1975; Wehner et al., 2000). Recognition impairment has been observed in previous studies that have described damage to the hippocampus as sufficient to create impairment of recognition memory (Broadbent et al., 2010).

Pole test measurements reflected the deterioration of motor function in the M/N-Day 5 but not in the M/N-Day 1 group. In the current study, the transient impairment of motor performance after methadone overdose suggests that perhaps alternative strategies with other brain regions involved in the processing of sensorimotor performance. Another explanation is, administration of naloxone (reversing methadone overdose) may partly reduce the motor disabilities following injection of a toxic dose of methadone with unknown mechanisms.

In the present study, the rotarod test for evaluation of motor coordination and balance showed mild or no deficiency in animals which experienced apnea. It is important to note that the lack of significant impairment in motor performance in the rotarod test could be in part due to the small number of rats which experienced apnea that had executed the test. It should be noted that other conditions such as test protocol, laboratory environmental factors, and rod diameter could have influenced the sensitivity of the test for detecting subtle deficiencies in motor function or balance following methadone-induced apnea. Nevertheless, several previous reports indicated the lack of motor coordination, executive function, and ataxia which were observed following methadone overdose (Tramullas et al., 2007; Cottencin et al., 2009).

The current results showed no changes in locomotor activity after a single toxic dose of methadone, which is inconsistent with the results of previous studies on the attenuating or increasing effect of motor activity following acute or chronic administration of methadone in rats (Mendez and Trujillo, 2008). Different routes of administration, patterns and doses of methadone prescribed, and the duration of recording of locomotion, as well as the different time point measurements, might affect the outcomes and produce different results (Allouche et al., 2013).

It also has been suggested that administration of naloxone after methadone overdose may modulate the detrimental effects of opioid receptor activation on both locomotor activity and motor coordination in the rotarod task. The results showed that administration of a single dose of naloxone had no effect on memory or motor performance. Hayward and Low reported that naloxone dose-dependently decreased motor activity, which is inconsistent with the current findings (Hayward and Low, 2005). It appears that the short duration of action of naloxone (Aghabiklooei et al., 2013) did not cause alterations in motor function at 24 h post-injection.

Administration of NMDA receptor antagonists like AP5 and MK-801 could impair spatial working memory. As a result, the antagonistic action of methadone on the NMDA receptors might confirm the hypothesis that methadone mediates through both opioid and NMDA receptors to exert adverse/neurotoxic effects on memory and motor function in different behavioral tasks (Ebert et al., 1995; Tsien et al., 1996; Nylander et al., 2016).

Delayed leukoencephalopathy was described for the first time after anoxic injury with symmetrical necrotic lesions of the central white matter, along with the damage to gray matter caused delayed neurological deterioration after initial recovery (Lin et al., 2012; Meyer, 2013). There are several reports of severe methadone-induced leukoencephalopathy which can be recognized by magnetic resonance imaging findings (Mittal et al., 2010; Cerase et al., 2011). The exact mechanism remains uncertain, but one possible hypothesis is that it is in part due to the defect in energy metabolism caused by demyelination following respiratory depression/arrest after methadone overdose (Weinberger et al., 1994). Direct damage or activation of immunological responses to brain tissue is another hypothesis which explains the pathogenesis of methadone-induced leukoencephalopathy (Mills et al., 2008; Mittal et al., 2010; Cerase et al., 2011; Rando et al., 2016). The serum half-life of morphine administered to an opioid-naïve patient was nearly 2–3 h, while this for methadone is approximately 150 h (Ciejka et al., 2016). With respect to methadone toxicity in cell culture, it has been suggested that methadone-induced cell death uncouples mitochondria, resulting in impairment of ATP synthesis (Perez-Alvarez et al., 2010; Nylander et al., 2016). Although these findings are not specific, such symptoms are in part consistent with the current behavioral results. Acute cerebellitis (Mills et al., 2008; Rando et al., 2016) or basal ganglia (Cottencin et al., 2009; Corliss et al., 2013) damage involvement following methadone overdose may explain the motor impairment observed in the pole and rotarod tasks caused in part by overstimulation of opioid receptors in these brain regions.

Despite its limitation, our findings indicate that following acute methadone overdose, reporting and follow-up assessment with the use of brain-imaging techniques after relative initial recovery should be performed. Utilization of plausible neurotoxicity biomarkers would allow continual monitoring to explore complications and possible damage to the central nervous system as well. It is suggested that methadone overdose should be considered to be a possible cause of delayed neurological disorders which require accurate monitoring for adverse reactions or signs to aid diagnosis of the risk of complications to the nervous system in hospital poison centers for healthy subjects, especially children. It has been noted that respiratory depression should be considered in patients using opioids for the first time, but not in chronic users because they develop a tolerance to opioid drugs. It has been suggested that overdose with long-acting opioids such as methadone by a naïve individual may require a longer observation period in the hospital to reduce delayed complications or possible long-term sequelae.

The important limitation of this study means, it remains unclear whether the impairments relate directly to methadone toxicity or cerebral hypoxia. Moreover, we did not measure the concentration of methadone in blood or brain tissue on test days due to the limited financial resource at the time of doing research.

## Conclusion

In contrast to the majority of studies on the neurological consequences of MMT patients, the current study has shown that acute exposure to a toxic dose of methadone in naïve healthy rats impaired cognitive and/or motor function. The deficient was reversible in motor function but not for memory during an observation period of nearly 2 weeks. The exact mechanisms remain uncertain, but further studies are required to elucidate the different pathophysiological mechanisms of methadone-induced neurotoxicity.

## Author Contributions

All authors designed the study, analyzed and interpreted the data, and wrote the paper.

## Conflict of Interest Statement

The authors declare that the research was conducted in the absence of any commercial or financial relationships that could be construed as a potential conflict of interest.
